# Disentangling boredom from depression using the phenomenology and content of involuntary autobiographical memories

**DOI:** 10.1038/s41598-024-52495-5

**Published:** 2024-01-24

**Authors:** Ryan C. Yeung, James Danckert, Wijnand A. P. van Tilburg, Myra A. Fernandes

**Affiliations:** 1 Department of Psychology, University of Waterloo, 200 University Avenue West, Waterloo, ON N2L 3G1 Canada; 2 Rotman Research Institute, Baycrest Health Sciences, 3560 Bathurst Street, Toronto, ON M6A 2E1 Canada; 3 Department of Psychology, University of Essex, Wivenhoe Park, Colchester, C04 3SQ UK

**Keywords:** Human behaviour, Neuroscience

## Abstract

Recurrent involuntary autobiographical memories (IAMs) are memories retrieved unintentionally and repetitively. We examined whether the phenomenology and content of recurrent IAMs could differentiate boredom and depression, both of which are characterized by affective dysregulation and spontaneous thought. Participants (*n* = 2484) described their most frequent IAM and rated its phenomenological properties (e.g., valence). Structural topic modeling, a method of unsupervised machine learning, identified coherent content within the described memories. Boredom proneness was positively correlated with depressive symptoms, and both boredom proneness and depressive symptoms were correlated with more negative recurrent IAMs. Boredom proneness predicted less vivid recurrent IAMs, whereas depressive symptoms predicted more vivid, negative, and emotionally intense ones. Memory content also diverged: topics such as relationship conflicts were positively predicted by depressive symptoms, but negatively predicted by boredom proneness. Phenomenology and content in recurrent IAMs can effectively disambiguate boredom proneness from depressive symptoms in a large sample of undergraduate students from a racially diverse university.

## Introduction

Boredom is a common human experience characterized as a negative affective state of wanting, but failing, to engage effectively with the world^1^. While theoretical accounts of in-the-moment feelings of boredom suggest that it functions as a self-regulatory signal prompting exploratory action^2^, the individual disposition for experiencing the state more frequently and intensely^3^ is associated with a raft of negative outcomes^4^.

Perhaps the most notorious mental health challenge associated with boredom proneness is depression^5^. A seminal finding in boredom proneness research, the relation with depression has since been shown to be moderate to strong in many large samples^5–7^. Despite these consistent associations between the two, prior research has shown that boredom proneness and depression are distinct affective experiences^8,9^. That is, boredom is not simply a milder form of depression, but represents a distinct state and trait disposition. As such, the nature of the relation between boredom and depression is an important one to disentangle, to determine whether and to what extent boredom and boredom proneness represent prodromal risk factors for the development of depression and other mental health challenges.

Both boredom proneness and depression share a wide range of characteristics, including negative affect^9^, attentional and memory difficulties^10,11^, low levels of self-reported arousal^12^, low self-control^13^, and a perceived lack of meaning in life^14,15^. These shared characteristics extend to behaviours such as withdrawal^16,17^, school and occupation absence^18,19^, substance use^20,21^, and impulsiveness^22,23^. These similarities suggest there may be common underlying experiences between boredom proneness and depression, including anhedonia and rumination. While anhedonia is best known for its core role in depressive disorders^24^, it has also been linked to boredom and boredom proneness^9^. Similarly, rumination features in both depression^25^ and boredom proneness^26^. Analogous to depressive rumination, wherein thought is passively focused on one’s circumstances and feelings without actively changing said circumstances^25^, state boredom is also characterized by a failure to launch into action—a feeling that one is stuck in an unsatisfying situation, fruitlessly ruminating on the need to act while failing to implement action^27^. Taken together, these findings suggest that boredom and boredom proneness may represent a precursor to depression^28^.

Boredom features a perceived lack of, and a desire to regain, meaning^14^ and challenge^29^. Boredom is one of the more prevalent negatively valenced emotions experienced across the lifespan^30^ and has been associated with situations involving low perceived challenge, meaninglessness, and difficulties with attentional control^8,10^. What might lead boredom to function as a precursor to depression? Bargdill^31^ suggests that habitual boredom (an analogue to trait boredom proneness^3^) arises when individuals feel that they have failed to successfully realize a large life goal. According to Bargdill^28^, more chronic experiences of boredom spread from a focus on a singular unrealized life goal, to affecting one’s life more broadly, leading to a more passive stance towards life. This in turn may lead to a common sense of hopelessness. Indeed, Farmer and Sundberg^5^, in developing the original Boredom Proneness Scale, characterized boredom prone individuals as people who “experience varying degrees of depression, hopelessness, (and) loneliness” (p. 14). Similarly, Tam and colleagues^32,33^ propose that the frequent and intense experience of boredom may develop into clinical issues through an operant conditioning process, where a lack of meaning and control become perceived as unchanging features of one’s life. These experiences are common to both chronic boredom and depression and may include a sense of futility in engaging with the world^28,34^. As such, boredom may set the scene for depression to arise by engaging self-evaluations of failure and meaninglessness, coupled with a sense of hopelessness in addressing these factors.

### Involuntary autobiographical memories in boredom and depression

Involuntary autobiographical memories (IAMs) may help disambiguate boredom proneness and depression. IAMs are memories of one’s personal past, retrieved unintentionally and effortlessly^35^. Past work shows that IAMs are sometimes experienced *recurrently*, such that memories of the same episode repeat involuntarily^36–38^. While these repetitions can range in frequency from several times per day to several times per year, the crux in this literature has been the subjective experience of the memory being repetitive^36^. Conceptual models of recurrent IAMs suggest that spontaneous cognitions of this kind act as a transdiagnostic mechanism of psychopathology^39,40^. In particular, recurrent IAMs and the experience of reliving vivid intrusive memories have been linked to depression^41^ and high levels of depressive symptoms^37,38,42^. The recurrence of intrusive, negatively valenced memories in depression may in part reflect one of the cardinal symptoms of the syndrome, namely rumination^25^: negative recurrent IAMs, and their strong impact on mood^43^, may initiate or maintain ruminative thought processes^44^. In addition, recurrent IAMs have been positively related to maladaptive coping strategies, including increased rumination and memory avoidance—both of which prolong depressive symptoms^45^. Thus, it is important to study IAMs in the context of mental health challenges and particular traits that may contribute to or exacerbate those challenges (e.g., boredom proneness).

While IAMs are relevant to psychopathology, they can also occur in response to nonpathological experiences. In this context, boredom proneness may be a particularly promising avenue. Participants frequently list boredom as an antecedent to IAMs^46^, and boredom proneness has been positively correlated with IAM frequency in daily life^47^. Additionally, a hallmark feature of boredom proneness is spontaneous mind wandering^13^. Spontaneous mind wandering itself is associated with poor self-regulation and cognitive inflexibility^44^, impaired memory^48^, and attention failures^1^. Importantly, mind wandering frequently involves episodic AMs^49^, suggesting that IAMs are a relevant experience in both depression and boredom proneness. What remains poorly understood is the actual *content* of spontaneous mind wandering or IAMs. Studying IAMs is thus a particularly promising way to gain insight into what goes on in the bored versus depressed mind; examining the content and nature of IAMs in the context of both boredom proneness and depression could illustrate what converges and diverges across these highly related, but distinct, experiences.

Given prior research demonstrating consistent relations between boredom proneness and depression^5,8^, here we thought it would be valuable to explore IAMs as a function of both. Having established that IAMs may be relevant for both depression and boredom proneness—empirically established for the former, theoretically plausible for the latter—it is possible to anticipate differences in their phenomenology and content. For example, while depression is especially characterized by negative thoughts in relation to past events [e.g., trauma^50^], those who are boredom prone likely experience thoughts about alternative activities and a desire for challenge, meaning, and excitement^51^. As such, it may well be that IAMs for the boredom prone differ from those related to depression, allowing for novel ways to identify whether one’s thought patterns are characteristic of being either boredom prone or depressed.

### Phenomenology and content in involuntary autobiographical memories

As already mentioned, prior work suggests that recurrent IAMs are transdiagnostic clinical features^39,40^, and as such, likely involved with a range of unpleasant emotional experiences (e.g., depression symptoms and boredom proneness). Recurrent IAMs could therefore illustrate how cognitive phenomena differ depending on one’s levels of boredom proneness and depressive symptoms. In particular, recurrent IAMs are known to vary along multiple dimensions, such as *phenomenology* (subjective experience of the memory) and *content* (what one reports remembering).

First, the phenomenology of autobiographical memory has been studied for decades^52^. Previous studies have highlighted the reliability and factor structure of properties of autobiographical memories^53^, which supports the validity of our measures and constrains our current work to a robust set of phenomenological variables. Further, research has broadly examined the phenomenological properties of IAMs experienced by those with major depressive disorder [e.g., vividness, level of distress, and associated kinetic sensations^54^]. We, too, have shown that phenomenology (e.g., self-reported valence) of recurrent IAMs is related to mental health status in both younger^37^ and older adults^38^. Here, we focused on the properties of frequency, vividness (perceived detail, visual imagery), valence, and emotional intensity. Prior research has highlighted these properties as relevant to depression, such that negative intrusive memories are experienced vividly by those with the disorder^45^. Boredom may have opposing relationships with these phenomenological properties: the highly boredom prone may fail to generate vivid internal stimuli^55^. In this manner, IAM phenomenology could help disentangle boredom proneness and depression given distinctions in their subjective experiences.

Second, there exists a long tradition of collecting and analyzing verbal descriptions of autobiographical memories^56^, with recent advances enabling large-scale content analysis in tandem with phenomenological variables^57^. Accordingly, some researchers have examined how the content of recurrent IAMs might distinguish depression from other disorders [i.e., PTSD^54,58^]. Past studies suggest that recurrent IAMs in depression are commonly associated with feelings of guilt, sadness, and anxiety^54^, and that thematically they can be divided into five broad domains: interpersonal issues, death/illness of a significant other, illness/injury to the self, personal assault/abuse, and other^58^. These findings suggest that content analysis of recurrent IAMs might offer unique insights when attempting to disentangle related constructs^59^. We recently applied computational methods to analyze content in thousands of participants’ IAMs, without the need to manually code each one^57^. Specifically, we used structural topic modeling^60^, a method of unsupervised machine learning, to extract topics [i.e., groups of words that can be interpreted as themes^61^] from participants’ text descriptions of their IAMs. The critical advantage of structural topic modeling versus other related techniques [e.g., latent Dirichlet allocation^61^] is that researchers can then analyze how variables of interest correlate with topics. Leveraging our previous approach is fruitful here, in that large-scale content analysis allows us to examine differences in memory content as a function of boredom proneness and depression symptoms. Where content characteristic of boredom proneness may be directed outwards—“The world is not enough”—content characteristic of depression symptoms may be directed inwards in a more self-critical manner^28^.

For these reasons, we believe that recurrent IAMs are both theoretically relevant (e.g., involved in both depression and boredom proneness) and practically useful (e.g., enabling measurement of phenomenology and content). As such, we suggest that recurrent IAMs represent an important opportunity to disambiguate the trait disposition of boredom proneness from the syndrome of depression. We hypothesized that recurrent IAMs would be experienced differently, or have distinct experiential foci, as a function of boredom proneness and depression symptoms. That is, if the phenomenology (subjective experience), as well as content (what people report remembering) of recurrent IAMs can reliably differentiate between boredom proneness and depression symptoms, this would provide insights into the association and distinction between the two. While clearly speculative at this point, this may in turn have implications for understanding the potential for boredom and boredom proneness to function as risk factors for mental health challenges including (but not restricted to) depression.

## Results

### Recurrent IAM presence

Participants who experienced recurrent IAMs within the past year (*n* = 3345) scored significantly higher for both depression symptoms (*M* = 6.34, *t*(6065.2) = − 9.58, *p* < 0.001, *d* = − 0.25) and boredom proneness (*M* = 27.36, *t*(6040.7) = − 5.69, *p* < 0.001, *d* = − 0.15), compared to those who had not experienced recurrent IAMs within the past year or ever (*n* = 2805, *M*_DASS-D_ = 5.08,* M*_SBPS_ = 26.02).

### Phenomenology

**Figure 1:**
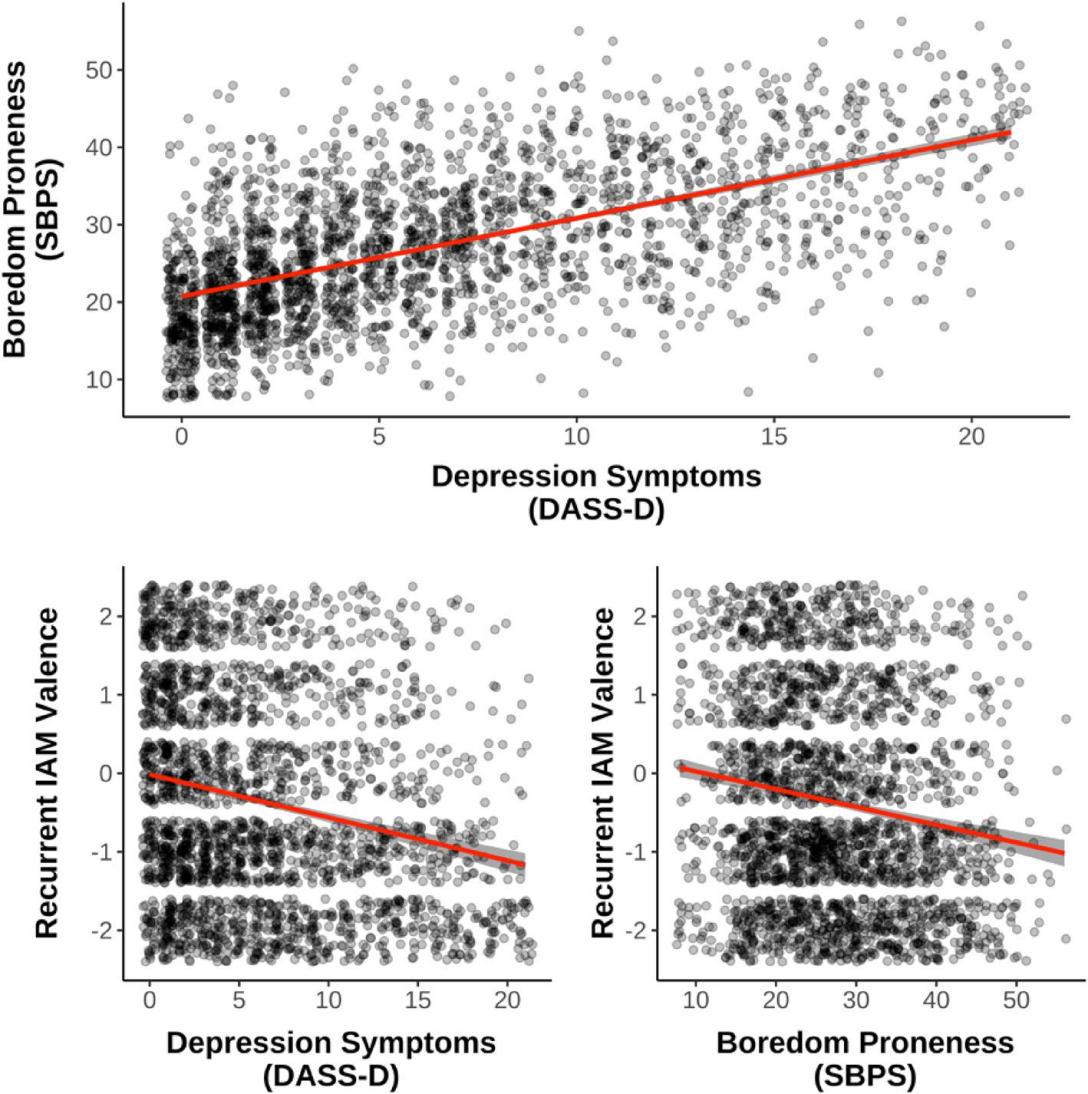
Correlations between boredom proneness, depression symptoms, and recurrent IAM valence. *DASS-D* Depression, Anxiety, and Stress Scales – Depression Subscale, *SBPS* Short Boredom Proneness Scale. Shaded ribbons represent 95% confidence intervals.

As expected, boredom proneness was significantly and positively correlated with depression symptoms (*r* = 0.58, *p* < 0.001). Further, both boredom proneness (*r* = − 0.16, *p* < 0.001) and depression symptoms (*r* = − 0.22, *p* < 0.001) were significantly correlated with recurrent IAM valence, such that higher scores on both traits were associated with more negative recurrent IAMs (Fig. 1; Table 1 shows the full correlation matrix with all variables).

**Table 1:** Correlation matrix of depression symptoms, boredom proneness, and recurrent IAM phenomenology.

Variable	1.	2.	3.	4.	5.	6.	7.
1. DASS-D	–						
2. SBPS	0.58***	–					
3. rIAM frequency	0.15***	0.08***	–				
4. rIAM detail	0.03	− 0.04	0.24***	–			
5. rIAM imagery	0.05*	− 0.02	0.18***	0.64***	–		
6. rIAM valence	− 0.22***	− 0.16***	− 0.17***	− 0.13***	− 0.02	–	
7. rIAM intensity	0.23***	0.14***	0.28***	0.35***	0.33***	− 0.44***	–

*DASS-D* Depression, Anxiety, and Stress Scales—Depression Subscale, *SBPS* Short Boredom Proneness Scale, *rIAM* recurrent involuntary autobiographical memory. ***p < 0.001, *p < 0.05.

**Figure 2:**
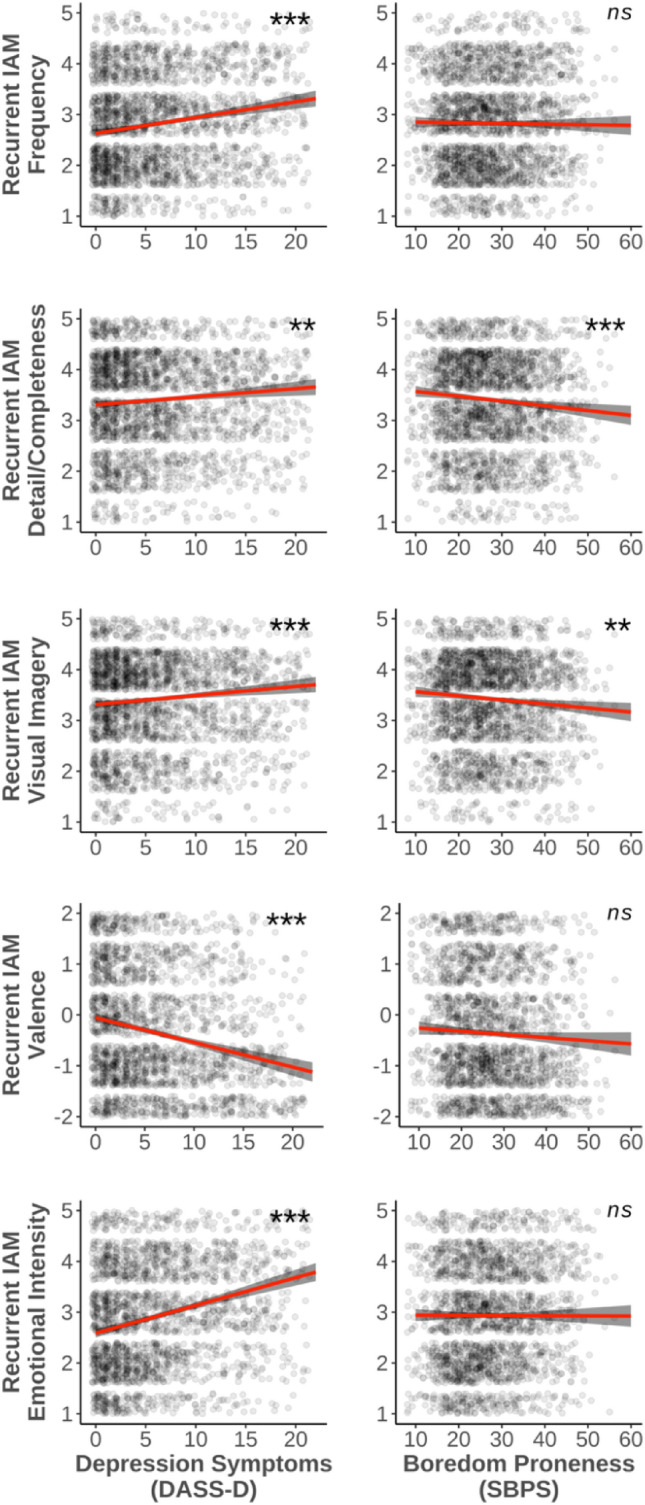
Partial effects of depression symptoms vs. boredom proneness on phenomenology of recurrent IAMs. Each row reflects a different regression model predicting a phenomenological property using depression symptoms and boredom proneness scores. Abbreviations as for Fig. 1. *p < 0.05, **p < 0.01, ***p < 0.001.

Multiple regressions were conducted for each phenomenological property of interest (i.e., frequency of recurring, detail/completeness, visual imagery, valence, emotional intensity) using depression symptoms and boredom proneness as predictors (Fig. 2). Predictors were entered simultaneously, with the phenomenological properties as outcome variables. Variance inflation factors were all less than 1.53, suggesting no issues related to multicollinearity.

Depression symptoms significantly predicted more frequent recurrence of IAMs (β = 0.16, *t*(2481) = 6.36, *p* < 0.001), whereas boredom proneness did not (β = − 0.001, *t*(2481) = − 0.45, *p* = 0.656; overall adjusted *R*^2^ = 0.02). Depression symptoms significantly predicted greater subjective feelings of detail/completeness (β = 0.08, *t*(2479) = 3.23, *p* = 0.001); in contrast, boredom proneness significantly predicted *lesser* subjective feelings of detail/completeness (β = − 0.08, *t*(2479) = − 3.31, *p* = 0.001; overall adjusted *R*^2^ = 0.005). Depression symptoms significantly predicted *stronger* visual imagery (β = 0.09, *t*(2480) = 3.74, *p* < 0.001), whereas boredom proneness significantly predicted *weaker* visual imagery (β = − 0.07, *t*(2480) = − 2.91, *p* = 0.004; overall adjusted *R*^2^ = 0.005). Depression symptoms significantly predicted more negative valence (β = − 0.20, *t*(2481) = − 8.09, *p* < 0.001), whereas boredom proneness did not (β = − 0.004, *t*(2481) = − 1.80, *p* = 0.07; overall adjusted *R*^2^ = 0.05). Finally, depression symptoms significantly predicted more emotional intensity (β = 0.24, *t*(2481) = 9.80, *p* < 0.001), whereas boredom proneness did not (β = − 0.001, *t*(2481) = − 0.10, *p* = 0.917; overall adjusted *R*^2^ = 0.05; Fig. 2).

Using the method recommended by [^62^; Eq. 4], slopes were significantly different between depression symptoms and boredom proneness for all phenomenological properties (*p*s < 0.001). These differences remained significant after using the Benjamini–Hochberg method^63^ of controlling the false discovery rate (q = 0.1).

### Content

#### Supervised dimension projection

**Figure 3:**
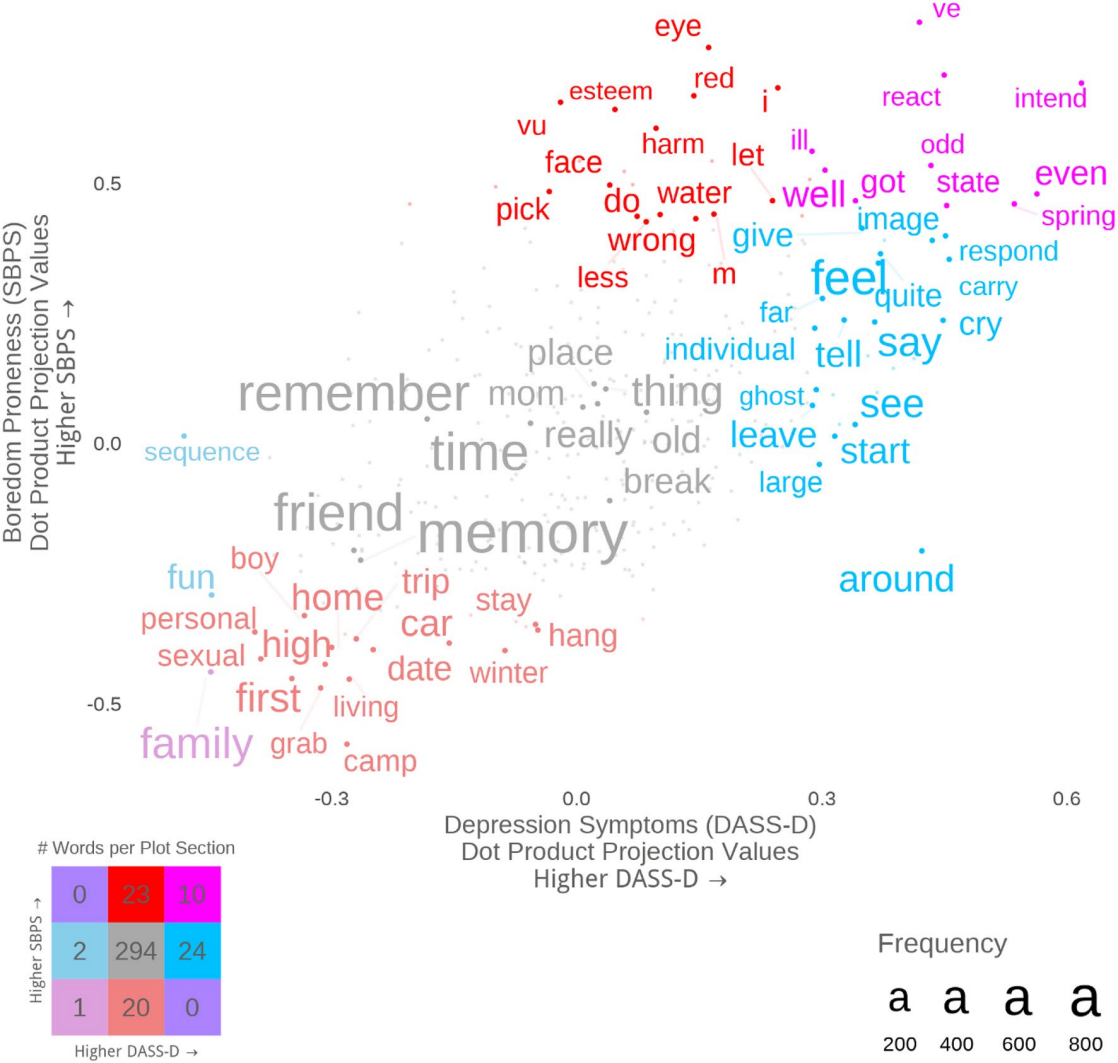
Supervised dimension projection plot of words in recurrent IAMs using depression symptoms and boredom proneness. Words in blue were most related to depression symptoms, whereas words in red were most related to boredom proneness. Words in pink were most related to both depression symptoms and boredom proneness, whereas words in grey were not significantly related to either. Note that some nonwords in this figure can be attributed to either contractions (e.g., “m” from “I’m”; “ve” from “I’ve”) or phrases (e.g., “vu” from “déjà vu”).

Figure 3 illustrates the words in recurrent IAMs most strongly related to depression symptoms and boredom proneness. Qualitatively, while words involving negative valence were strongly related to high scores on both constructs, there appeared to be more internally focused content in high depression symptoms (e.g., “feel”, “cry”) versus externally focused content in high boredom proneness (e.g., “harm”, “wrong”).

#### Topic modeling

The final topic structure obtained is shown in Table 2. The goal of these researcher-assigned labels was simply to facilitate interpretation of content by topic.

**Table 2:** Topics in recurrent IAMs.

Topic number	Researcher-assigned label	Top ten most representative words (based on FREX scores)	Topic prevalence (%)
1	Embarrassing, unsettling events	Dream, thing, may, experience, mostly, situation, person, actually, wish, environment	8.4
2	Salient travel events	Walk, ride, snow, night, home, sister, bike, panic, sunny, dad	5.5
3	Childhood friendships	High, song, elementary, school, listen, play, old, hang, friend, tv	7.7
4	Car accidents	Car, pass, accident, crash, drive, young, funeral, away, age, two	4.9
5	Nostalgic trips	Recollection, trip, time, frequent, spend, country, meet, conversation, recurrently, every	7.9
6	Family holidays	Childhood, memory, many, together, social, consist, relate, recollect, group, topic	6.8
7	Regrets	Someone, embarrassed, think, people, like, question, flashback, now, make, differently	6.9
8	Distressing relationship conflicts	Relationship, ex, assault, sexually, traumatic, fail, bad, partner, feeling, argument	7.8
9	Vivid, cued, negative events	Something, embarrassing, sometimes, usually, detail, vivid, specific, trigger, can, affect	7.8
10	Deaths	Send, parent, death, cry, hospital, brother, goodbye, mental, health, mom	6.3
11	Stress and performance	Abuse, anxiety, job, university, trauma, ago, bully, conflict, term, emotional	5.8
12	Positive events	Event, certain, fun, life, random, involve, occur, different, head, pop	7.4
13	Classroom experiences	Teacher, door, girl, grade, middle, class, chair, classmate, inside, classroom	6.8
14	Pleasant family gatherings	Eat, child, win, dinner, vacation, water, cousin, table, game, buy	6.1
15	Aggressive confrontations	Angry, move, birthday, another, dog, kind, roommate, work, argue, card	3.8

*FREX* frequency-exclusivity^87^.

#### Predicting topic prevalence

**Figure 4:**
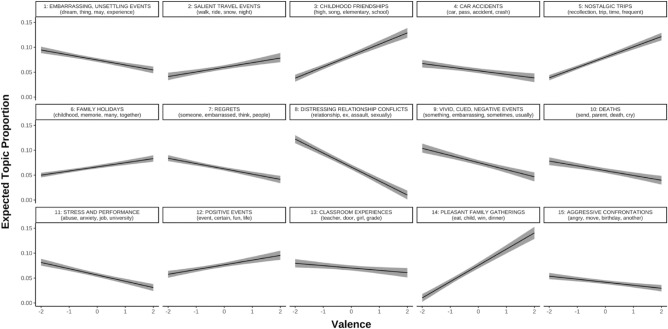
Predicted topic prevalence using valence. Valence of recurrent IAMs was self-reported (-2 = very negative, 2 = very positive). Valence was a significant predictor for all topics (*p*s < 0.02).

First, to confirm the validity of the researcher-assigned labels, we estimated topic prevalence using self-reported IAM valence as a predictor. Valence was a significant predictor for all topics (*p*s < 0.02); qualitatively, positive labels were given to topics predicted by positive valence and vice versa (Fig. 4).

**Figure 5:**
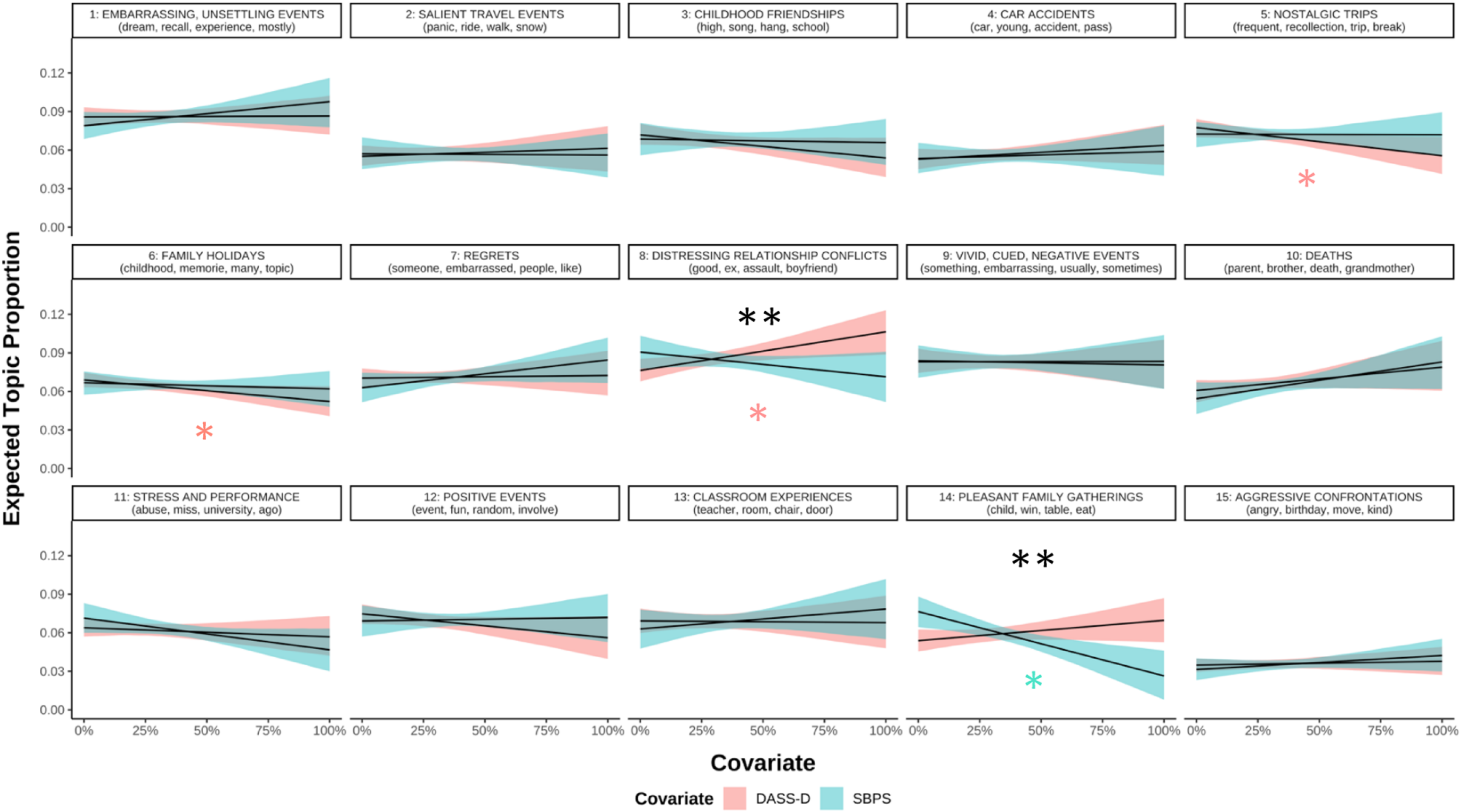
Predicted topic prevalence using depression symptoms and boredom proneness. *DASS-D* Depression, Anxiety, and Stress Scales—Depression Subscale, *SBPS* Short Boredom Proneness Scale. For this figure, DASS-D and SBPS scores were converted to percent of maximum possible scores (POMP; Cohen et al., 1999) so that units could be compared on the same scale (i.e., 0% = lowest possible score on either measure, 100% = highest possible score). Coloured asterisks refer to significant predictors. Black asterisks refer to models where slopes significantly differed from each other. *p < 0.05, **p < 0.01.

Second, to examine whether content changed as a function of depression and boredom proneness, we estimated topic prevalence using depression symptoms and boredom proneness as predictors. Critically, depression symptoms and boredom proneness explained unique variance in several topics (Fig. 5). Depression symptoms were significantly predictive of greater use of topic 8 (“Distressing relationship conflicts”; B = 0.001, *p* = 0.01), and less use of topic 5 (“Nostalgic trips”; B = − 0.001, *p* = 0.03) and topic 6 (“Family holidays”; B = − 0.0007, *p* = 0.04). Boredom proneness was significantly predictive of less use of topic 14 (“Pleasant family gatherings”; B = − 0.001, *p* < 0.001).

Using methods recommended by [^62^; Eq. 4], regression coefficients were significantly different between depression symptoms and boredom proneness for topics 8 (“Distressing relationship conflicts”; *p* = 0.005) and 14 (“Pleasant family gatherings”; *p* = 0.006). These differences remained significant after using the Benjamini–Hochberg method^63^ of controlling the false discovery rate (q = 0.1). Slopes were not significantly different for any other topic (*p*s > 0.065).

As an exploratory analysis to control for valence, we also estimated topic prevalence with depression symptoms, boredom proneness, and valence as predictors (see Supplemental Materials). Results were similar to the previous model (depression symptoms and boredom proneness as predictors, without valence). As in the previous model, depression symptoms were significantly predictive of greater use of topic 8 (“Distressing relationship conflicts”; B = 0.001, *p* = 0.02), but also less use of topic 9 (“Vivid, cued, negative events”; B = − 0.001, *p* = 0.03) and greater use of topic 14 (“Pleasant family gatherings”; B = 0.001, *p* = 0.049). Inconsistent with the previous model (without valence), boredom proneness was significantly predictive of less use of topic 8 (“Distressing relationship conflicts”; B = − 0.0008, *p* = 0.01). As with the previous model (without valence), slopes were significantly different between depression symptoms and boredom proneness for topics 8 (“Distressing relationship conflicts”; *p* = 0.005) and 14 (“Pleasant family gatherings”; *p* = 0.006), even after applying the same Benjamini–Hochberg corrections^63^. Slopes were not significantly different for any other topic (*p*s > 0.051).

## Discussion

We investigated whether the related constructs of boredom proneness and depression symptoms could be distinguished using the phenomenology and content of recurrent IAMs. Though we did not directly examine the role of demographics on the patterns we report, our sample was derived from data collected at a large Canadian university, consisting of undergraduate students from racially diverse backgrounds. Results showed that phenomenology and content in recurrent IAMs can effectively disambiguate boredom proneness from depression symptoms.

Prior research suggests that boredom and depression are distinct affective experiences^8,9^, yet has left largely unanswered how they differ. Documenting and differentiating relations between these two affective states is important. First, boredom and depression share a large number of problematic correlates, such as substance use^20,21^, poorer diet and exercise^64^, social withdrawal and loneliness^65^, problem gambling^66^, and even suicidal ideation^67^. Combatting these problematic outcomes requires a nuanced understanding of their causes.

It is of theoretical importance to understand better the differences between boredom and depression. A hallmark feature of both is that they involve an acute lack of perceived meaning in life or one’s activities^14,51^. The question remains as to why the lack of meaning in some contexts lead to boredom, and in others depression. Boredom and depression are well-established correlates^5,6^, which some have interpreted to reflect the possibility that chronic boredom may function as a potential precursor or risk factor for depression, for example when boredom is left to fester unresolved^28^. By understanding the differences between the two, it becomes possible to identify what marks the point at which boredom turns, over time, into depression.

With respect to phenomenology of IAMs, boredom and depression showed some distinct patterns. While both showed negative relations with valence, such that higher trait boredom or depressive symptoms were associated with more negatively valenced IAMs (Fig. 1), there were notable differences in other domains. Namely, boredom proneness was not significantly predictive of either the frequency or emotional intensity of experiencing IAMs (Fig. 2), whereas higher levels of depressive symptoms were associated with more frequent and emotionally intense IAMs. Although preliminary, this may reflect one avenue by which boredom proneness differs from depressive symptoms—a simple increase in the frequency and intensity of negatively valenced IAMs appears to be characteristic of depression rather than boredom proneness. Critically, boredom proneness and depressive symptoms had opposing relationships with vividness-related memory qualities, with boredom proneness being associated with a lack of detail and visual imagery, and depression symptoms being associated with more perceived detail and imagery (Fig. 2). This suggests a kind of bland or generic nature to the IAMs experienced by highly boredom prone people; memories that, while recurrent, lack the intensity and complexity of memories experienced by those lower in boredom proneness. Greater perceived detail and imagery with higher depression symptoms also aligns with previous findings, wherein individuals with depression reported their negative intrusive memories as being highly vivid^68^. Finally, we also observed that depression symptoms were significantly related to a wider range of properties than boredom proneness. It would appear that depression symptoms tended to explain more variance in recurrent IAM phenomenology compared to boredom proneness, which is perhaps unsurprising given the long literature on altered autobiographical memory processes in depression^45,50^. Though here we focus on the fact that slopes significantly differed for depression symptoms and boredom proneness, future work could explore their relative shares of variance explained.

Content likely plays a role, too. Qualitatively, words associated with high boredom proneness appeared to be more externally focused and related to actions (“do”, “harm”, “wrong”), whereas words associated with depression symptoms appeared to be more internally focused, emotional, and social/communicative (“feel”, “cry”, “say”; Fig. 3). This pattern seems to converge with previous work finding positive correlations between depressive symptoms and episodic details (e.g., emotions felt, persons present) in nonclinical samples^69–71^. To explore content with greater granularity, we used computational text analysis, and showed that boredom proneness and depressive symptoms uniquely predicted the prevalence of different topics in recurrent IAMs. In line with the suggestion that IAMs for the highly boredom prone were somewhat bland, the trait negatively predicted the prevalence of two topics: distressing relationship conflicts and (in the exploratory analysis controlling for valence) pleasant family gatherings. These could be seen as two ends of an affective spectrum, and for the highly boredom prone, neither seems to be a common topic in recurrent IAMs. In contrast, depressive symptoms positively predicted the prevalence of the distressing relationship conflicts topic (Fig. 5). This topic frequently contained descriptions of abuse and may highlight a common proximal correlate of depression.

Interestingly, based on the exploratory analysis controlling for valence (see Supplemental Materials), depressive symptoms also negatively predicted the prevalence of content related to vivid, cued, negative events. At first blush, this seems contradictory to the positive relation between depression symptoms and vividness, as well as the negative relation between depression symptoms and valence (Fig. 2). It may be the case that people with higher depression symptoms feel an event’s emotions intensely, but are less likely to describe it as such when asked (e.g., providing details could itself be triggering of the negative affective experience)^72^. In addition, it may be the case that individuals with high depressive symptoms are less likely to recollect specific details of memories (as instructed to do here) compared to those with low depressive symptoms. This suggestion is in line with recent work showing that depression is characterized by reduced detail generation when recalling specific events from one’s personal past^73,74^. So, while those experiencing high depressive symptoms may report recurrent IAMs as being intense, they may be less able (or less willing) to outline the specific content when not explicitly instructed to do so^69,72^. An interesting point here is that previous studies have observed this pattern with deliberate recall of (hypothetically) voluntary AMs^73,74^; while the current study also involved deliberate recall (following^36,37,75^), participants recounted/rated memories that they had previously experienced *involuntarily*. Although the correspondence between the current and past results is encouraging, future studies could directly compare AMs along the voluntary-involuntary spectrum to shed light on how these different retrieval types relate to depression and boredom proneness.

In sum, the current evidence supports many existing ideas in the literature, including that (a) boredom proneness and depression symptoms are related, but separable constructs, and (b) that recurrent IAMs are associated with a variety of (often unpleasant) affective experiences. Critically, our work advances these ideas by pinpointing specific dimensions along which boredom proneness and depression symptoms have differing, or even opposing, relations. Our findings suggest that the ‘objectless’ nature of boredom proneness can manifest as recurrent IAMs that are less vivid and less likely to be about affectively intense events (e.g., pleasant family gatherings). On the other hand, depression symptoms can manifest as recurrent IAMs that are more frequent, vivid, more likely about negative social events (e.g., distressing relationship conflicts) and less likely about positive social events (e.g., nostalgic trips, family holidays). While boredom proneness and depression symptoms may overlap in many ways, individuals’ memories (e.g., how they are subjectively experienced or reconstructed) can highlight differences in the inner workings of the bored versus depressed mind.

A number of limitations for the current study are also worth noting. First, depression symptoms as measured by the DASS-D (in the past week) may not have aligned temporally with the experience of any given recurrent IAM. Interestingly, our data still suggest that there are consistent relations between recurrent IAMs and depression symptoms despite the scales not being temporally aligned. This suggests that these relationships could be more trait-like or dispositional rather than being dependent on any specific episode of depressive symptoms evoking specific IAMs (or vice versa). Additionally, the correlational nature of this study and its measures means that the current study does not provide causal or directional evidence. Future work could examine the temporal dynamics or directionality of recurrent IAMs, boredom proneness, and depressive symptoms using experience sampling methods.

The present study also only measured participants’ most frequently recurring IAMs (following^36,37,75^). As such, the recurrent IAM reported by the participant may not be the *only* one they have. Furthermore, while we asked for their most frequently recurring one, it is possible that participants recalled their most salient (or otherwise accessible) IAM. While our procedure matches prior work^36,37^ to maintain comparability, this also means that our current findings apply only to participants’ most frequently recurring IAMs, and do not speak to the importance of less frequent ones, nor the relevance of having multiple recurrent IAMs. Future research could determine whether the phenomenology or content of multiple recurrent IAMs within an individual could further differentiate boredom proneness and depression.

We also limited our current analyses to main effects of boredom proneness and depression symptoms. This was motivated by our research questions, which concerned the independent effects of the two variables, and to what extent they converged or diverged in relation to recurrent IAMs. To follow up on the present findings, further studies could examine interactions between boredom proneness and depression symptoms. That is, additional work could investigate whether the observed relationships between recurrent IAMs and boredom proneness also change as a function of depression symptoms (and vice versa).

Another general note is that there is ongoing scholarly discussion about the measurement and divergent validity of boredom proneness and depressive symptoms. While boredom proneness has been consistently positively associated with symptoms of depression, at least one study has used structural equation modeling to convincingly show that the two things are distinct^9^. Nevertheless, other work has raised concerns regarding just what the Boredom Proneness Scale (and by extension the Short Boredom Proneness Scale) measures^76^. Future work could make use of more recent measures that overcome these concerns (e.g.,^77^) to further parse the distinctions between boredom and symptoms of depression.

Overall, we have shown that the phenomenology and content of one’s memories, as reflected in recurrent IAMs, can effectively disambiguate boredom proneness and depression symptoms. By comprehensively examining recurrent IAMs in a large sample, the current study identified specific axes upon which cognition varies as a function of boredom proneness versus depressive symptoms (e.g., external vs. internal focus, vividness, emotional intensity). Our work may open avenues for practitioners to distinguish boredom proneness more easily from depression symptoms, and to examine the circumstances that may explain the relation between the two.

## Methods

We report how we determined our sample size, all data exclusions, all manipulations, and all relevant measures in the study.

### Participants

Over two years (Sept. 2018–Jan. 2020), 6187 unique undergraduate students who were enrolled in at least one psychology course at the University of Waterloo self-registered for our online study. Participants completed a battery of questionnaires in a randomized order, including the Recurrent Memory Scale, in which they indicated whether they had experienced any recurrent IAMs (i.e., personal memories retrieved unintentionally and repetitively) within the past year. If so, they described their most frequently recurring IAM in text and rated its phenomenology (e.g., frequency of recurring, detail/completeness, visual imagery, valence, and emotional intensity). Participants also completed self-report scales measuring trait boredom proneness and depression symptoms, along with other measures unrelated to the current study. Subsets of the current dataset were previously published in^37,42,57^.

Our final sample consisted of the 2484 participants who had experienced recurrent IAMs within the past year (*n*_excluded_ = 2805), had provided valid text descriptions of recurrent IAMs (i.e., excluding those who wrote meaningless or irrelevant text, using the detection process described in^78^; *n*_excluded_ = 142), and had no missing responses on the scales of interest (e.g., depression symptoms, boredom proneness, recurrent IAM description or phenomenology; *n*_excluded_ = 956). Note that participants could have met multiple exclusion criteria (e.g., wrote invalid text and provided no response for at least one item in a scale of interest). Notably, these exclusion rates are quite typical based on prior studies. Past work has shown that approximately 50% of large, nonclinical samples report having not experienced recurrent IAMs in the past year, or ever^36–38^. The number of invalid texts was also comparable to past studies^37,57,78^. Finally, cases where no response was provided were typical, especially considering that participants tend to skip open text items^79^.

In our final sample, 71% of participants were women, 28% were men, and 1% were nonbinary, genderqueer, or gender nonconforming. The mean age was 19.9 years (*SD* = 3.3, range = 16–49). Participants were mostly White/Caucasian (39%), East Asian (23%), or South Asian (19%), and were primarily born in Canada (66%), China (9%), or India (5%; see Supplemental Materials for full breakdown). All procedures were approved by the University of Waterloo’s Office of Research Ethics (Protocol #40049) and informed consent was obtained prior to completing any of the scales. Data and code supporting the results of this study are openly available at: 10.17605/OSF.IO/83Y2R.

### Materials

#### Recurrent memory scale

We used the Recurrent Memory Scale^37^ to assess participants’ recurrent IAMs. Participants indicated if they had experienced at least one recurrent IAM within the past year, not within the past year, or never, as instructed by the following prompt adapted from^36^:

Sometimes, people experience that memories from their personal past may come to mind by themselves. That is, the memory seems to spontaneously pop into mind, effortlessly and without having tried to remember it. Do you experience that the same recollections recurrently pop into your mind by themselves—so that memories for the same event repeat themselves in consciousness? We are not asking about dreams, but about recollections that you experience when you are awake^37^.

If they had experienced at least one within the past year, they wrote a brief description of their most frequently recurring IAM (*M*_word count_ = 32.3, *SD*_word count_ = 22.2), as instructed by the following prompt: “Please briefly describe your memory of the event in 3–5 sentences, without any identifying information”^37^. Following the description, they estimated how long ago the original event occurred (*M*_years ago_ = 3.8, *SD*_years ago_ = 4.9) and rated the memory’s phenomenology on a series of 5-point Likert scales. Of interest in the current study were the items measuring frequency of recurring (“How often within the most recent year have you experienced that same recollection returning to your thoughts by itself?”; 1 = *only once*, 5 = *several times a day*), detail/completeness (“How complete and detailed is your memory for the event?”; 1 = *not complete or detailed*, 5 = *very complete and detailed*), vividness of visual imagery (“If you experience visual images of the event when remembering it, how vivid are these images?”; 1 = *cloudy or no image at all*, 5 = *as vivid as normal vision*), valence (“Is the recollection emotionally very positive, positive, neutral, negative, or very negative?”; -2 = *very negative*, 2 = *very positive*), and emotional intensity (“Is the recollection emotionally not at all intense, a little intense, somewhat intense, intense, or very intense?”; 1 = *not at all intense*, 5 = *very intense*).

#### Depression anxiety stress scales

We administered the DASS-21^80^ to assess depression symptoms, as indexed by the 7-item depression subscale (DASS-D). Participants indicated the degree to which the items applied to them over the past week. Internal consistency was high in the current sample for the full scale (α = 0.96), as well as the depression subscale (α = 0.94).

#### Short boredom proneness scale

We administered the SBPS^7^ to assess boredom proneness, which consists of eight items indexing the tendency to experience boredom. Internal consistency was high in the current sample (α = 0.88).

### Text analysis

#### Supervised dimension projection

We first sought to provide a visual depiction of memory content as a function depression symptoms and boredom proneness. To do so, we constructed a supervised dimension projection plot^81^ for participants’ text descriptions of their recurrent IAMs. In brief, this method uses word embeddings to identify words that are significantly related to high versus low scorers on variables of interest (i.e., depression symptoms and boredom proneness). Text data were tokenized, cleaned (e.g., punctuation removal), and lemmatized before we constructed word embeddings using the pretrained RoBERTa model.

For visual inspection purposes, we also created word clouds of terms used relatively more frequently by those high in depression symptoms versus those high in boredom proneness (i.e., those scoring in the top tertile of one, but not the other; Fig. S2), or those high in both (i.e., those scoring in the top tertile of both; Fig. S3; see Supplemental Materials).

#### Topic modeling

To formally analyze content of recurrent IAMs beyond the level of single words, we then used structural topic modeling^60^—a method of unsupervised machine learning—to identify topics in participants’ memories and examine the unique relationships between these topics and both boredom proneness and depression symptoms. Topic modeling also offers benefits in terms of word sense disambiguation or polysemy (words having the same spelling but different meanings, e.g., “bat” as in “baseball bat” versus “fruit bat”), since words can be assigned to different topics if they consistently appear in different contexts^82^.

Typical topic modeling procedures are reported elsewhere in detail^57^. To summarize, we applied standard preprocessing steps^83,84^, including tokenization, cleaning (e.g., removing punctuation), stop word removal (using the Snowball stop word list), vocabulary pruning (excluding words that appeared in fewer than three documents across the entire corpus), and lemmatization. Researchers must also select an a priori number of topics (i.e., groups of words that can be interpreted as themes)^60,61^ to be identified when using structural topic modeling^60^. To select an appropriate number of topics, we simulated and inspected models with the same parameters (e.g., covariates) across a varying number of topics (i.e., between 5 and 25 topics). We then selected an appropriate number of topics using a two-stage approach^85^.

**Figure 6:**
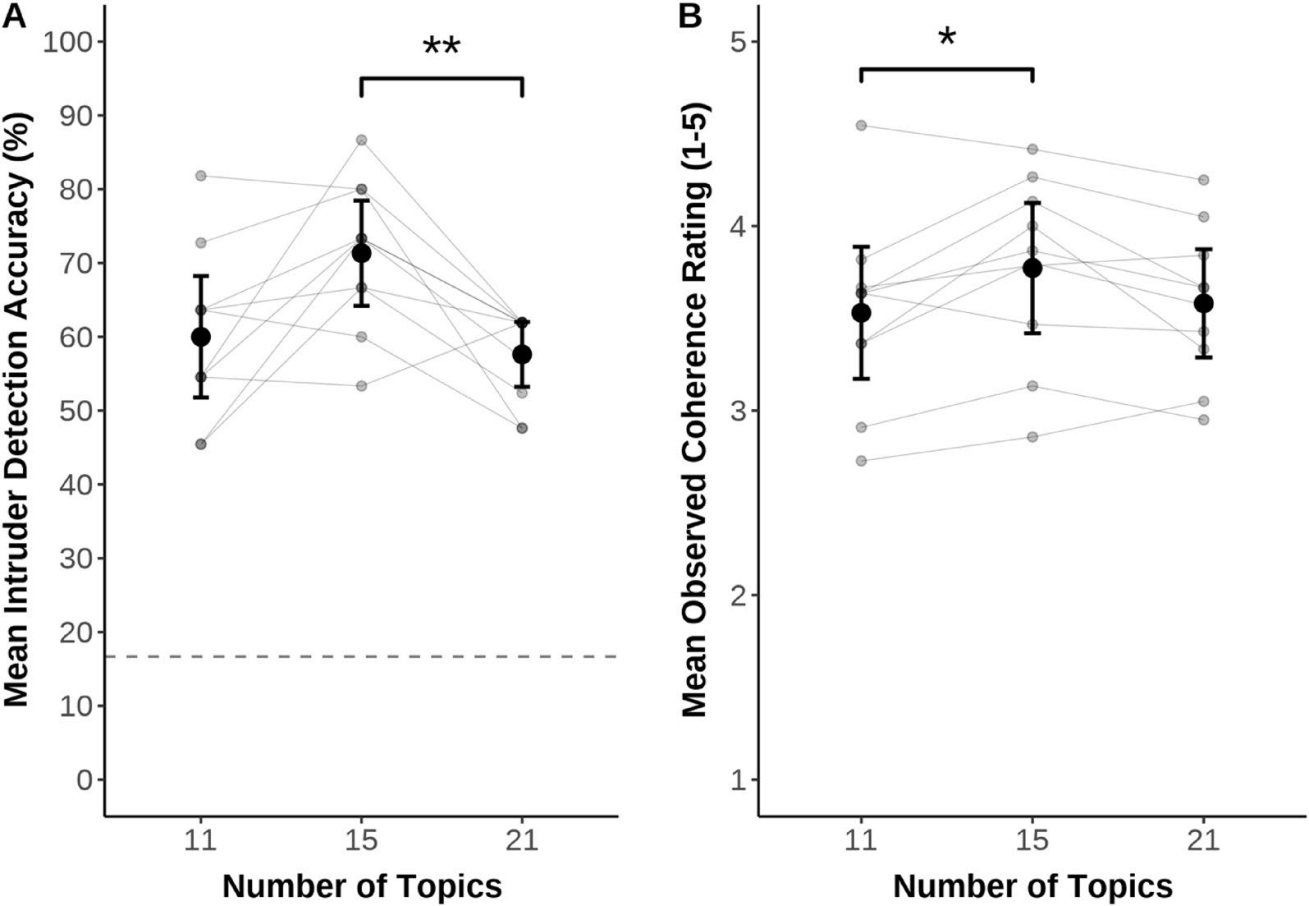
Accuracy and observed coherence in word intrusion task by topic number. Responses during the word intrusion task, separated into intruder detection accuracy (**A**) and observed coherence ratings (**B**). Error bars represent 95% confidence intervals. Dashed line in (**A**) indicates chance performance. Grey points indicate individual participants’ data, with grey lines between points indicating data points from the same participants. *p < 0.05, **p < 0.01. All other comparisons were nonsignificant (*p*s > 0.051).

First, internal validation (based on computed metrics derived from the data) guided the initial selection of candidate models. Following^60^, metrics were computed for each possible model (i.e., number of topics), including semantic coherence (degree to which words *within a topic* co-occur more so than words *across different topics*)^86^, exclusivity (degree to which words are specific to few topics versus general across many topics)^87^, and held-out likelihood (“probability of words appearing within a document when those words have been removed from the document in the estimation step”)^88^ (p. 38). Local maxima for semantic coherence, exclusivity, and held-out likelihood values were found at 11, 15, and 21 topics; accordingly, we chose these three as candidate models (Fig. 6).

Second, external validation (based on human judgment and performance measures) guided the selection of the final model from the three candidate models. This entailed administering a word intrusion task^89^ to ten independent raters who were naïve to the goals and hypotheses of the study (all raters were research assistants in the labs of authors JD, WvT, and MF). On each trial, raters saw sets of six words. Unbeknownst to the raters, five of the words were highly representative (highest five frequency-exclusivity scores)^87^ of one topic (e.g., “car, “pass”, “accident”, “crash”, “drive”), while the remaining word was highly representative (sixth highest frequency-exclusivity score) of a *different*, randomly selected topic (e.g., “class”). Raters were asked to identify the word that did not belong (i.e., the intruder or word from the different topic), with successful intruder detection being interpreted as better model quality^83^. Following this judgment, raters were shown the five words belonging to the same topic and rated the degree to which they seemed to cohere (i.e., observed coherence; from 1 = *very incoherent* to 5 = *very coherent*)^89^. Comparing intrusion detection accuracy and observed coherence across the three candidate models (11, 15, 21 topics), ANOVAs and post hoc Tukey tests indicated preference for the 15-topic model as the final model (Fig. 6; also see Supplemental Materials).

A deliberation process was then conducted to generate researcher-assigned labels characterizing each topic in the final model^57,84,85^. Each author was provided with the top ten most representative words for each topic, twenty documents (i.e., IAMs reported by participants) predicted to contain the highest proportions of each topic, and other internal, computationally derived metrics (e.g., coherence, exclusivity). Using this information, all authors generated labels independently before meeting to agree on labels. Agreement on the independently generated labels was high; all 15 topics were independently assigned labels that matched across at least 50% of the authors, even before discussion (100% agreement for nine topics, 75% agreement for four topics, 50% agreement for two topics; Table 2).

### Supplementary Information

Supplementary Information.

## Data Availability

Data and code supporting the results of this study are openly available at: https://doi.org/10.17605/OSF.IO/83Y2R.
