# Supplementary material for: Disentangling boredom from depression using the phenomenology and content of involuntary autobiographical memories
Source: Sci Rep. 2024 Jan 24;14:2106. doi: 10.1038/s41598-024-52495-5 (PMC10808106; doi:10.1038/s41598-024-52495-5)
Supplement: Supplementary file 1 — Supplementary Information. [file 41598_2024_52495_MOESM1_ESM.docx]

Supplemental Materials for:

**Disentangling boredom from depression using the phenomenology and content of involuntary autobiographical memories**

Ryan C. Yeung, James Danckert, Wijnand A. P. van Tilburg, & Myra A. Fernandes

**External Validation of Topic Models**

Intruder detection accuracy and observed coherence scores were compared across the three candidate models (11, 15, 21 topics) using ANOVAs, both of which were significant (*p*s < .01). Post hoc Tukey tests indicated significantly greater intruder detection accuracy with the 15-topic model versus the 21-topic model (*p* = .008), and significantly greater observed coherence with the 15-topic model versus the 11-topic model (*p* = .04); no other differences were significant (*p*s > .051).

As an exploratory analysis, we repeated the within-subjects comparison of observed coherence ratings after scaling and centering this variable at the participant level. Results were similar to the unstandardized data, in that the ANOVA was significant (*F*(2, 18) = 4.31, *p* = .03). The post hoc Tukey tests were also similar and in the same directions as the unstandardized data, but the difference between 11 and 15 topics was no longer significant (*p* = .056; Figure S1).

**Figure S1**

*Standardized Observed Coherence in Word Intrusion Task by Topic Number*


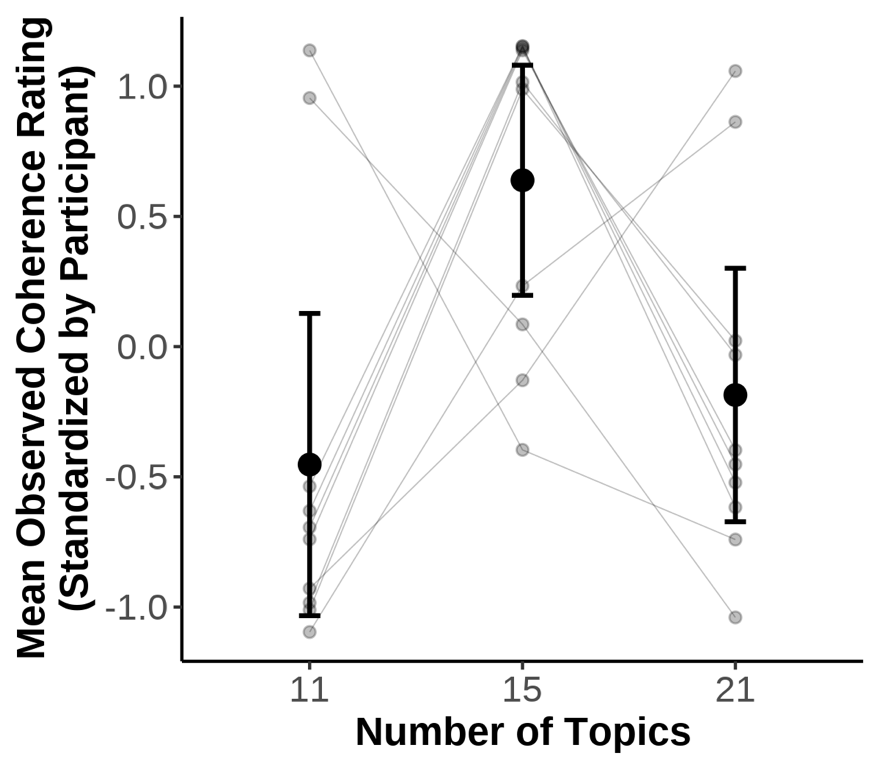


*Note.* Observed coherence ratings were standardized within each participant for this analysis/figure. Error bars represent 95% confidence intervals. Grey points indicate individual participants’ data, with grey lines between points indicating data points from the same participants. All comparisons were nonsignificant (ps > .056).

**Table S1**

*Self-Reported Primary Racial Group*

| **Primary Racial Group** | ***n* (%)** |
| --- | --- |
| Aboriginal (First Nations) | 11 (0.18%) |
| Black/African | 188 (3.04%) |
| East Asian (e.g., Chinese, Japanese, Korean) | 1408 (22.76%) |
| Hispanic | 97 (1.57%) |
| Middle Eastern | 238 (3.85%) |
| Mixed | 139 (2.25%) |
| South Asian (e.g., Pakistani, Indian) | 1151 (18.60%) |
| Southeast Asian (e.g., Filipino, Vietnamese, Indonesian) | 309 (4.99%) |
| West Indian/Caribbean | 106 (1.71%) |
| White/Caucasian | 2384 (38.53%) |
| [Decline to Answer] | 100 (1.62%) |
| Other not listed above | 56 (0.91%) |

*Note.* Instructions for this item were as follows: “What is your primary racial group? If you identify with more than one group, please select one group that represents an important identity for you.”

**Table S2**

*Self-Reported Ethnic Identity*

| **Ethnic Identity** | ***n* (%)** |
| --- | --- |
| Aboriginal (North America First Nations, Inuit, or Métis) | 8 (0.13%) |
| African | 69 (1.12%) |
| American | 35 (0.57%) |
| Arab | 75 (1.21%) |
| Bangladesh | 29 (0.47%) |
| Black/African | 29 (0.47%) |
| Black/African-American | 5 (0.08%) |
| Black/African-Canadian | 74 (1.2%) |
| Brazilian | 2 (0.03%) |
| British/English | 48 (0.78%) |
| Bulgarian | 2 (0.03%) |
| Cambodian | 10 (0.16%) |
| Canadian | 2671 (43.17%) |
| Cantonese | 179 (2.89%) |
| Chinese | 576 (9.31%) |
| Croatian | 8 (0.13%) |
| Czech | 2 (0.03%) |
| Danish | 1 (0.02%) |
| Dutch | 22 (0.36%) |
| Egyptian | 41 (0.66%) |
| European | 42 (0.68%) |
| Filipino | 92 (1.49%) |
| Finnish | 2 (0.03%) |
| French | 8 (0.13%) |
| French-Canadian | 33 (0.53%) |
| German | 24 (0.39%) |
| Greek | 20 (0.32%) |
| Guyanese | 14 (0.23%) |
| Hispanic | 26 (0.42%) |
| Hongkongese | 34 (0.55%) |
| Hungarian | 5 (0.08%) |
| Indian (from India) | 490 (7.92%) |
| Indonesian | 14 (0.23%) |
| Iranian | 26 (0.42%) |
| Iraqi | 8 (0.13%) |
| Irish | 23 (0.37%) |
| Israeli | 5 (0.08%) |
| Italian | 71 (1.15%) |
| Jamaican | 29 (0.47%) |
| Japanese | 14 (0.23%) |
| Latin-American | 30 (0.48%) |
| Lithuanian | 1 (0.02%) |
| Malaysian | 9 (0.15%) |
| Maltese | 2 (0.03%) |
| Mennonite | 21 (0.34%) |
| Middle Eastern | 14 (0.23%) |
| Norwegian | 1 (0.02%) |
| Other not listed | 197 (3.18%) |
| Pakistani | 147 (2.38%) |
| Palestinian | 10 (0.16%) |
| Persian | 9 (0.15%) |
| Polish | 27 (0.44%) |
| Portuguese | 39 (0.63%) |
| Romanian | 18 (0.29%) |
| Russian | 18 (0.29%) |
| Scandinavian | 3 (0.05%) |
| Scottish | 28 (0.45%) |
| Serbian | 21 (0.34%) |
| Slovak | 2 (0.03%) |
| South Korean | 96 (1.55%) |
| Spanish | 10 (0.16%) |
| Sri Lankan | 134 (2.17%) |
| Swedish | 1 (0.02%) |
| Swiss | 2 (0.03%) |
| Taiwanese | 28 (0.45%) |
| Trinidadian | 17 (0.27%) |
| Ukrainian | 10 (0.16%) |
| Vietnamese | 95 (1.54%) |
| Welsh | 3 (0.05%) |
| West Indian/Caribbean | 34 (0.55%) |
| Yugoslavian | 7 (0.11%) |
| N/A | 1 (0.02%) |
| [Decline to Answer] | 286 (4.62%) |

*Note.* Instructions for this item were as follows: “Which specific ethnic/cultural group listed below do you MOST identify with? This is not an exhaustive list - it provides responses that have been given previously by students. If your ethnicity is not accurately represented here, please specify below.”

**Table S3**

*Self-Reported Birth Country*

| **Birth Country** | ***n* (%)** |
| --- | --- |
| Australia | 6 (0.1%) |
| Austria | 2 (0.03%) |
| Bangladesh | 26 (0.42%) |
| Belarus | 1 (0.02%) |
| Belgium | 2 (0.03%) |
| Belize | 1 (0.02%) |
| Bosnia and Herzegovina | 2 (0.03%) |
| Brazil | 2 (0.03%) |
| Bulgaria | 1 (0.02%) |
| Burma | 1 (0.02%) |
| Canada | 4060 (65.62%) |
| Chile | 2 (0.03%) |
| China | 528 (8.53%) |
| Colombia | 11 (0.18%) |
| Congo | 1 (0.02%) |
| Costa Rica | 1 (0.02%) |
| Croatia | 1 (0.02%) |
| Cuba | 2 (0.03%) |
| Denmark | 1 (0.02%) |
| Dominican Republic | 1 (0.02%) |
| Egypt | 44 (0.71%) |
| El Salvador | 3 (0.05%) |
| Ethiopia | 9 (0.15%) |
| Finland | 1 (0.02%) |
| France | 8 (0.13%) |
| Germany | 22 (0.36%) |
| Greece | 4 (0.06%) |
| Hong Kong | 67 (1.08%) |
| Hungary | 1 (0.02%) |
| India | 335 (5.41%) |
| Indonesia | 16 (0.26%) |
| Iran | 29 (0.47%) |
| Iraq | 17 (0.27%) |
| Ireland | 2 (0.03%) |
| Israel | 4 (0.06%) |
| Italy | 3 (0.05%) |
| Jamaica | 17 (0.27%) |
| Japan | 6 (0.1%) |
| Kenya | 20 (0.32%) |
| Macao | 1 (0.02%) |
| Malaysia | 9 (0.15%) |
| Mexico | 5 (0.08%) |
| Moldova | 1 (0.02%) |
| Netherlands | 6 (0.1%) |
| New Zealand | 2 (0.03%) |
| Nicaragua | 1 (0.02%) |
| Nigeria | 42 (0.68%) |
| Norway | 2 (0.03%) |
| Other African country | 42 (0.68%) |
| Other Asian country | 99 (1.6%) |
| Other Australasian / Oceanian country | 1 (0.02%) |
| Other European country | 10 (0.16%) |
| Other North American country | 3 (0.05%) |
| Other South American country | 16 (0.26%) |
| Other West Indian country | 7 (0.11%) |
| Pakistan | 113 (1.83%) |
| Panama | 1 (0.02%) |
| Peru | 3 (0.05%) |
| Philippines | 53 (0.86%) |
| Poland | 5 (0.08%) |
| Portugal | 1 (0.02%) |
| Romani | 5 (0.08%) |
| Romania | 8 (0.13%) |
| Russia | 16 (0.26%) |
| Saudi Arabia | 26 (0.42%) |
| South Africa | 6 (0.1%) |
| South Korea | 104 (1.68%) |
| Spain | 2 (0.03%) |
| Sri Lanka | 19 (0.31%) |
| Sudan | 2 (0.03%) |
| Sweden | 1 (0.02%) |
| Switzerland | 5 (0.08%) |
| Syria | 9 (0.15%) |
| Taiwan | 26 (0.42%) |
| Thailand | 3 (0.05%) |
| Trinidad and Tobago | 13 (0.21%) |
| Turkey | 14 (0.23%) |
| UK | 39 (0.63%) |
| Ukraine | 3 (0.05%) |
| USA | 125 (2.02%) |
| Venezuela | 6 (0.1%) |
| Vietnam | 21 (0.34%) |
| Yugoslavia | 4 (0.06%) |
| N/A | 1 (0.02%) |
| [Decline to Answer] | 46 (0.74%) |

*Note.* Instructions for this item were as follows: “In which country/region were you born?”

**Table S4**

*Predicted Topic Prevalence Using Depression Symptoms and Boredom Proneness*

| **Predictor** | **Topic Number** | **B** | **SE B** | ***p*** |
| --- | --- | --- | --- | --- |
| DASS-D | 1 | < .0001 | 0.0005 | 0.939 |
| SBPS | 1 | 0.0004 | 0.0003 | 0.186 |
| DASS-D | 2 | 0.0003 | 0.0006 | 0.612 |
| SBPS | 2 | < .0001 | 0.0003 | 0.950 |
| DASS-D | 3 | -0.0008 | 0.0005 | 0.0855 |
| SBPS | 3 | -0.0001 | 0.0003 | 0.860 |
| DASS-D | 4 | 0.0005 | 0.0005 | 0.340 |
| SBPS | 4 | 0.0001 | 0.0003 | 0.726 |
| DASS-D | 5 | -0.0010 | 0.0005 | 0.0341 |
| SBPS | 5 | < .0001 | 0.0003 | 0.977 |
| DASS-D | 6 | -0.0008 | 0.0004 | 0.0374 |
| SBPS | 6 | -0.0001 | 0.0002 | 0.679 |
| DASS-D | 7 | 0.0001 | 0.0006 | 0.903 |
| SBPS | 7 | 0.0005 | 0.0003 | 0.130 |
| DASS-D | 8 | 0.0014 | 0.0006 | 0.0125 |
| SBPS | 8 | -0.0004 | 0.0003 | 0.201 |
| DASS-D | 9 | -0.0001 | 0.0006 | 0.824 |
| SBPS | 9 | < .0001 | 0.0003 | 0.967 |
| DASS-D | 10 | 0.0009 | 0.0006 | 0.180 |
| SBPS | 10 | 0.0006 | 0.0003 | 0.0676 |
| DASS-D | 11 | -0.0003 | 0.0005 | 0.495 |
| SBPS | 11 | -0.0005 | 0.0003 | 0.0699 |
| DASS-D | 12 | -0.0009 | 0.0005 | 0.103 |
| SBPS | 12 | 0.0001 | 0.0003 | 0.856 |
| DASS-D | 13 | -0.0001 | 0.0006 | 0.914 |
| SBPS | 13 | 0.0003 | 0.0004 | 0.417 |
| DASS-D | 14 | 0.0007 | 0.0006 | 0.186 |
| SBPS | 14 | -0.0011 | 0.0003 | 0.001 |
| DASS-D | 15 | 0.0001 | 0.0004 | 0.709 |
| SBPS | 15 | 0.0002 | 0.0002 | 0.290 |

*Note.* DASS-D = Depression, Anxiety, and Stress Scales – Depression Subscale; SBPS = Short Boredom Proneness Scale.

**Table S5**

*Predicted Topic Prevalence Using Depression Symptoms, Boredom Proneness, and Recurrent IAM Valence*

| **Predictor** | **Topic Number** | **B** | **SE B** | ***p*** |
| --- | --- | --- | --- | --- |
| DASS-D | 1 | -0.0003 | 0.0005 | 0.498 |
| SBPS | 1 | 0.0004 | 0.0003 | 0.128 |
| Valence | 1 | -0.0102 | 0.0015 | < .001 |
| DASS-D | 2 | 0.0004 | 0.0005 | 0.443 |
| SBPS | 2 | 0.0003 | 0.0003 | 0.348 |
| Valence | 2 | 0.0093 | 0.0016 | < .001 |
| DASS-D | 3 | -0.0001 | 0.0005 | 0.851 |
| SBPS | 3 | 0.0002 | 0.0003 | 0.521 |
| Valence | 3 | 0.0221 | 0.0018 | < .001 |
| DASS-D | 4 | 0.0002 | 0.0006 | 0.741 |
| SBPS | 4 | -0.0001 | 0.0003 | 0.781 |
| Valence | 4 | -0.0073 | 0.0019 | < .001 |
| DASS-D | 5 | 0.0001 | 0.0004 | 0.750 |
| SBPS | 5 | -0.0002 | 0.0002 | 0.482 |
| Valence | 5 | 0.0207 | 0.0014 | < .001 |
| DASS-D | 6 | -0.0004 | 0.0004 | 0.247 |
| SBPS | 6 | < .0001 | 0.0002 | 0.971 |
| Valence | 6 | 0.0086 | 0.0011 | < .001 |
| DASS-D | 7 | -0.0002 | 0.0004 | 0.593 |
| SBPS | 7 | 0.0002 | 0.0002 | 0.444 |
| Valence | 7 | -0.0104 | 0.0014 | < .001 |
| DASS-D | 8 | 0.0014 | 0.0006 | 0.0204 |
| SBPS | 8 | -0.0008 | 0.0003 | 0.0146 |
| Valence | 8 | -0.0284 | 0.0019 | < .001 |
| DASS-D | 9 | -0.0012 | 0.0005 | 0.0260 |
| SBPS | 9 | < .0001 | 0.0003 | 0.880 |
| Valence | 9 | -0.0139 | 0.0018 | < .001 |
| DASS-D | 10 | 0.0007 | 0.0005 | 0.220 |
| SBPS | 10 | 0.0005 | 0.0003 | 0.106 |
| Valence | 10 | -0.0095 | 0.0016 | < .001 |
| DASS-D | 11 | -0.0006 | 0.0004 | 0.185 |
| SBPS | 11 | -0.0004 | 0.0003 | 0.134 |
| Valence | 11 | -0.0123 | 0.0016 | < .001 |
| DASS-D | 12 | -0.0008 | 0.0005 | 0.147 |
| SBPS | 12 | 0.0001 | 0.0003 | 0.741 |
| Valence | 12 | 0.0097 | 0.0017 | < .001 |
| DASS-D | 13 | 0.0002 | 0.0006 | 0.702 |
| SBPS | 13 | 0.0001 | 0.0003 | 0.847 |
| Valence | 13 | -0.0051 | 0.0020 | 0.0119 |
| DASS-D | 14 | 0.0011 | 0.0006 | 0.0498 |
| SBPS | 14 | -0.0006 | 0.0003 | 0.0657 |
| Valence | 14 | 0.0326 | 0.0019 | < .001 |
| DASS-D | 15 | -0.0005 | 0.0004 | 0.265 |
| SBPS | 15 | 0.0004 | 0.0002 | 0.0624 |
| Valence | 15 | -0.0061 | 0.0012 | < .001 |

*Note.* DASS-D = Depression, Anxiety, and Stress Scales – Depression Subscale; SBPS = Short Boredom Proneness Scale.

**Table S6**

*Normality of Variables*

| **Variable** | **Skew** | **Kurtosis** |
| --- | --- | --- |
| DASS-D | 0.92 | 0.01 |
| SBPS | 0.35 | -0.36 |
| rIAM Frequency | 0.16 | -0.77 |
| rIAM Detail | -0.29 | -0.67 |
| rIAM Imagery | -0.37 | -0.54 |
| rIAM Valence | 0.40 | -0.98 |
| rIAM Intensity | 0.09 | -1.03 |

*Note.* DASS-D = Depression, Anxiety, and Stress Scales – Depression Subscale; SBPS = Short Boredom Proneness Scale; rIAM = recurrent involuntary autobiographical memory.

**Figure S2**

*Word Cloud of Recurrent IAMs for Individuals with High Depression Symptoms vs. High Boredom Proneness*


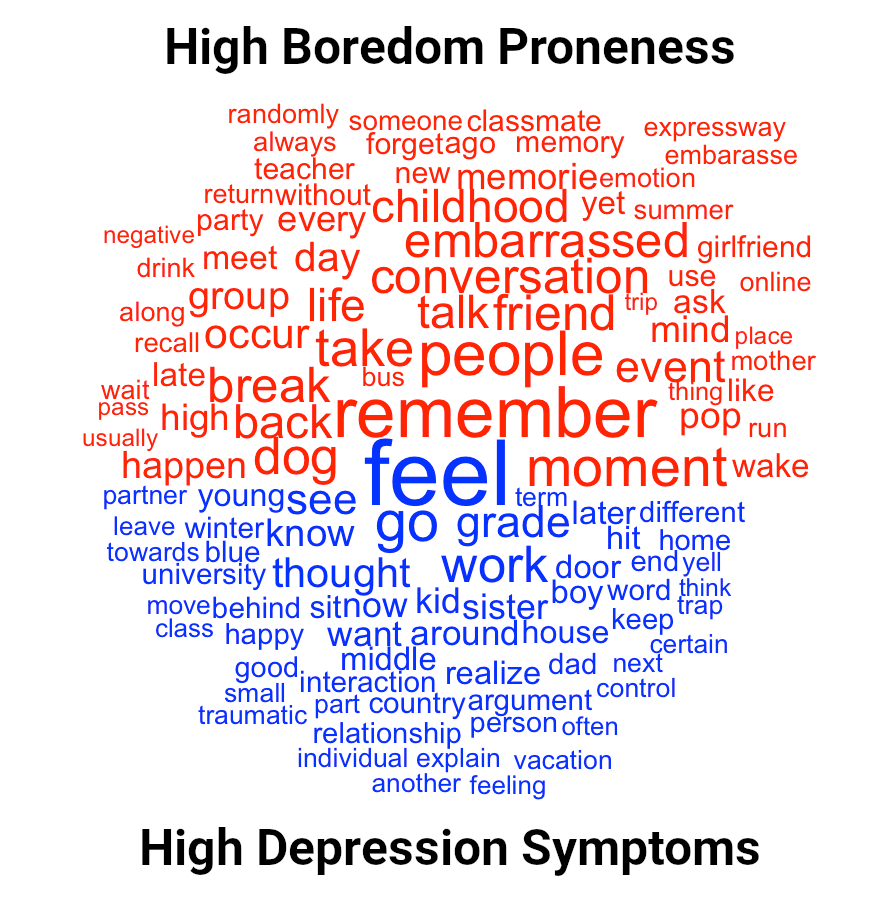


*Note*. Larger font size denotes more frequent occurrences of a term. Red font indicates greater relative occurrences of a term in the high boredom proneness group; blue font indicates greater relative occurrences of a term in the high depression symptoms group. High boredom proneness was defined as scoring within the highest tertile of boredom proneness (and *not* the highest tertile of depression symptoms); high depression symptoms was defined as scoring within the highest tertile of depression symptoms (and *not* the highest tertile of boredom proneness).

**Figure S3**

*Word Cloud of Recurrent IAMs for Individuals with High Depression Symptoms, High Boredom Proneness, or High Scores on Both*


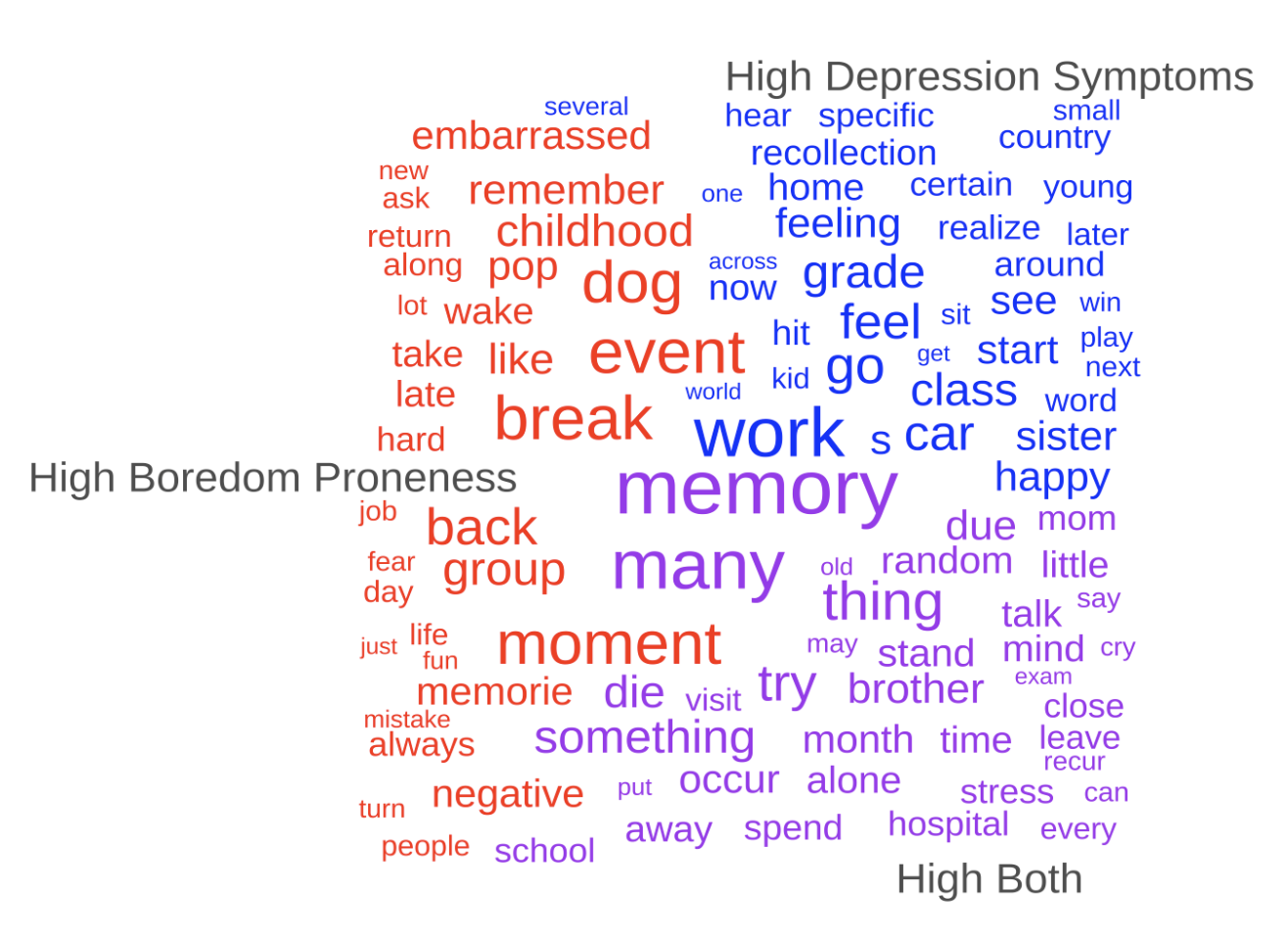


*Note*. Larger font size denotes more frequent occurrences of a term. Red font indicates greater relative occurrences of a term in the high boredom proneness group; blue font indicates greater relative occurrences of a term in the high depression symptoms group; purple font indicates greater relative occurrences of a term in the high both group. High boredom proneness was defined as scoring within the highest tertile of boredom proneness (and *not* the highest tertile of depression symptoms); high depression symptoms was defined as scoring within the highest tertile of depression symptoms (and *not* the highest tertile of boredom proneness); high both was defined as scoring within the highest tertile of boredom proneness *and* the highest tertile of depression symptoms.
